# Serum adenosine deaminase activity and acute cerebral infarction: a retrospective case-control study based on 7913 participants

**DOI:** 10.18632/aging.204338

**Published:** 2022-10-17

**Authors:** Yanyan Ling, Chuan Jiang, Zhenzhen Xiao, Xiao Shang, Qi Li, Baojie Wang, Maolin Hao, Fei Liu, Nannan Zhao, Jianli Feng, Hongqin Zhao

**Affiliations:** 1 Department of Neurology, Shandong Second Provincial General Hospital, Jinan 250000, China; 2 Department of Neurology, The Affiliated Hospital of Qingdao University, Qingdao 266000, China

**Keywords:** adenosine deaminase, acute cerebral infarct, uric acid, adenosine

## Abstract

Background: Adenosine deaminase (ADA) is a key enzyme that catalyzes the deamination of adenosine into inosine, which eventually decomposes into uric acid (UA). A body of papers have reported that adenosine and UA are closely related to cerebrovascular events. However, the association between serum ADA activity and acute cerebral infarction (ACI) remains unclear.

Methods: 7913 subjects were enrolled, including 3968 ACI patients and 3945 controls, in this study. An automatic biochemistry analyzer was used to determine serum activity.

Results: Serum ADA activity was found that was significantly decreased in patients with ACI (10.10 ± 3.72 U/L) compared to those without ACI (11.07 ± 2.85 U/L, *p* < 0.001). After Logistic regression analysis, ADA concentrations were negatively correlated with ACI (OR = 1.161, 95% CI: 1.140–1.183, *p* < 0.001). Smoking and alcohol consumption decreased serum ADA concentrations in patients with ACI, whereas diabetes and hypertension had the opposite effect.

Conclusions: Serum ADA concentrations in patients with ACI are markedly decreased, suggesting that the decreased ADA concentrations may be involved in the pathogenesis of ACI. We hypothesized that decreased ADA activity may be an adaptive mechanism to maintain adenosine levels and protect against ischemic brain injury.

## INTRODUCTION

Adenosine deaminase (ADA) is a 41 kDa monomer protein whose functions included not only catalyst activity, co-stimulatory, allosteric modifications, and cell-cell communication, but it also plays a vital role in purine metabolism [1]. The enzyme is generally distributed in human tissues such as the thymus and spleen, with the highest levels found in the gastrointestinal tract and moderate activity found in the brain [2]. Deficiency of ADA activity can lead to severe combined immunodeficiency which manifested as liver disease, tuberculosis, infectious mononucleosis, HIV infection, and reperfusion injury of the infracted myocardium [3, 4].

With the development of medical technology, the survival expectancy of patients with ACI has greatly improved, but it is still a major threat to human health [5]. Adenosine has been known as a neuroprotective agent for more than 30 years. Phillis and his colleagues [6] showed that the neuroprotective mechanisms include inhibition of neuronal excitability through adenosine, reducing intracellular calcium levels, and reducing nerve damage. Notably, adenosine may be an important endogenous neuroprotectant [6–8]. Extracellular concentrations of adenosine can increase from normal baseline levels of approximately 1 mM to 100 mM or more during and after hypoxic or ischemic attacks [9]. Moreover, elevated adenosine levels after ischemic stroke has been reported [10, 11]. However, there are few reports on the association between ADA activity and ACI occurrence. This study aim was to explore this relationship.

## METHODS

### Study population

The study enrolled a total of 7913 participants in the Affiliated Hospital of Qingdao University from December 2012 to June 2019, including 3968 subjects who met the diagnostic criteria for ACI and 3945 controls. Patients with a diagnosis of ACI were supported by magnetic resonance imaging (MRI) findings. The study excluded (1) individuals who underwent incomplete laboratory tests; (2) individuals with autoimmune diseases, such as systemic lupus erythematosus, liver disease, blood diseases, tuberculosis, and other serious illnesses; and (3) individuals with hemorrhagic stroke. The 3945 participants in the control group had no signs and symptoms of ACI, and MRI did not support the diagnosis. The written consent of all subjects or their legal representatives has been obtained. The study was approved by the Ethics Committee of the Affiliated Hospital of Qingdao University.

### Clinical parameters

A detailed medical history and risk factors for ACI were recorded for all participants. Height and weight were recorded in centimeters (cm) and kilograms (Kg) respectively. Body mass index (BMI) was calculated as weight (kg) divided by the square of height (m). Systolic and diastolic blood pressure were measured twice every 30 minutes with an automatic oscilloscope device, and the mean values of systolic and diastolic blood pressure were taken respectively. Hypertension was diagnosed when systolic blood pressure (≥140 mmHg) and/or diastolic blood pressure (≥90 mmHg), or using antihypertensive medications. Diabetes mellitus (DM) was diagnosed if hypoglycemic drugs were used or fasting blood glucose (FBG) level ≥7.0 mmol/L or glycosylated hemoglobin (HbA1c) concentration ≥6.5%.

### Evaluation of intracranial arterial stenosis

All subjects with ACI in the study underwent three-dimensional time-of-flight (3D TOF) magnetic resonance angiography (MRA) (3D- TOF MRA) with 3.0 T magnetic resonance scans. We defined intracranial vascular stenosis using the Warfarin-Aspirin Symptomatic Intracranial Disease (WASID) test criteria [12]. Intracranial artery stenosis (ICAS) was diagnosed when MRA showed occlusion or 50% to 99% stenosis. The following vessels were evaluated: bilateral internal carotid artery (ICA), bilateral middle cerebral artery (MCA, M1/M2), anterior cerebral artery (ACA, A1/A2), posterior cerebral artery (PCA, P1/P2), vertebral artery (VA), or basilar artery (BA).

### Biochemical measurements

Blood samples from all participants were taken after overnight fasting of at least 8 hours. Whole blood samples from participants were collected by vacuum tube in the absence of anticoagulant and centrifuged at 1500 × g for 15 min. Serum concentrations of alanine aminotransferase (ALT), total cholesterol (TC), low--density lipoprotein cholesterol (LDL-C), serum creatinine (SCr), triglycerides (TG), high-density lipoprotein cholesterol (HDL-C), fasting blood glucose (FBG), UA, and ADA was measured with an automatic biochemistry analyzer (Hitachi HCP-7600, Hitachi, Japan).

### Adenosine deaminase assay

**Figure 1:**
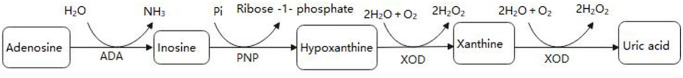
The enzymatic reaction scheme.

ADA concentrations were tested at 37°C based on Giusti and Galanti [13]. ADA activity was measured by the peroxidase method using a commercial kit (Beijing Leadman Biochemistry Co., Ltd; China). ADA enzyme deaminates adenosine to generate inosine. Inosine generates hypoxanthine through purine nucleoside phosphorylase (PNP). The latter yielded UA and hydrogen peroxide (H_2_O_2_) under xanthine oxidase (XOD). Finally, H2O2 was also reacted with N-Ethyl-N- (2-hydroxy-3-sulfopropyl) -3-methylaniline and 4-aminoantipyrine in the presence of peroxidase to generate quinone dye that is monitored kinetically. Under standard assay conditions, the amount of enzyme required for adenosine to release 1 mmol of ammonia per minute was defined as 1 unit (1U) of ADA. Figure 1 shows the process of the enzymatic reaction.

### Statistical analyses

SPSS statistical software was used for statistical analysis (version 25.0; SPSS Inc., Chicago, Illinois, USA). The mean ± standard deviation (SD) was used to describe continuous data, while frequency and percentage were used to describe categorical variables. For comparisons in categorical variables, we used the Chi-square test. The study variables were compared between the patient group and the control group using the unpaired student’s *t*-test, and Spearman’s correlation coefficients was used to assess interrelationships. To investigate the interaction of other variables between ADA concentrations and ACI, logistic regression was used in this study. A two-sided test was used for statistical analysis, and *P* < 0.05 were considered statistically significant.

## RESULTS

**Figure 2:**
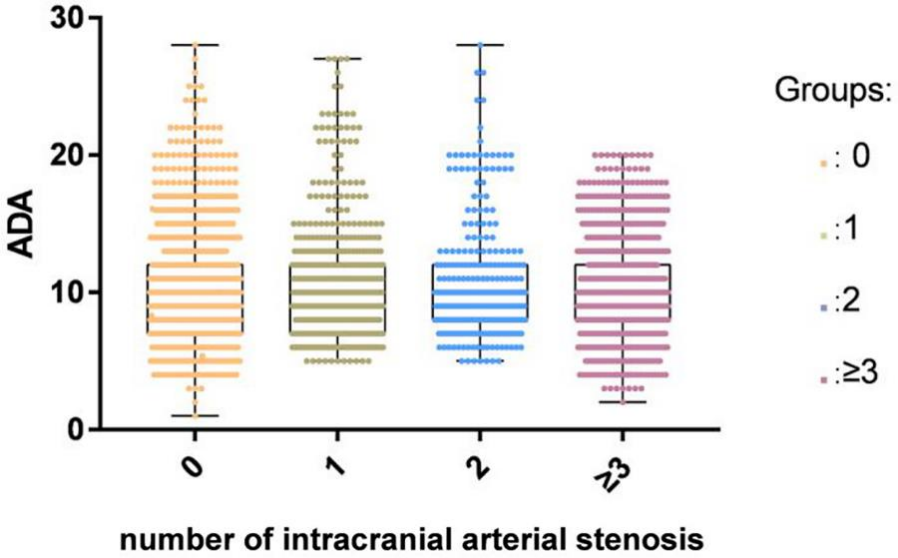
There was no significant difference in ADA activity among different ICAS groups classified by the number of stenotic arteries in the ACI group.

A total of 7913 participants were enrolled including 3968 patients with ACI (68.33 ± 11.08 years) and 3945 controls without ACI (60.79 ± 14.51years). Of the 3968 ACI patients, 536 patients had single-diseased blood vessels, 313 patients presented two-diseased blood vessels and 1290 had three-diseased blood vessels. The concentrations of TG, BMI, FBG, TC, LDL-C, and ALT in the ACI group were higher than those in the control group. Patients with ACI were significantly older than those in the control group. Furthermore, the rates of hypertension, DM, smoking, and alcohol consumption were higher in the patient group than in the control group. We observed no statistical difference in SCr between the control group and the patients. There was no significant difference in ADA activity between different ICAS groups classified by the number of stenotic arteries (Figure 2). The information and clinical characteristics of the participants are shown in Table 1.

**Table 1:** Demographic and clinical characteristics of ACI patients and controls.

**Variable**	**ACI (3968)**	**Non-ACI (3945)**	***P* value**
Age, years^*^	68.33 ± 11.08	60.79 ± 14.51	<0.001
Gender, male *n* (%) ^#^	2779 (70.04%)	2161 (54.78%)	<0.001
BMI (kg/m^2^) ^*^	25.36 ± 5.69	24.99 ± 8.49	0.023
Hypertension, *n* (%) ^#^	2573 (64.84%)	879 (22.28%)	<0.001
Diabetes, *n* (%) ^#^	1087 (27.39%)	128 (3.24%)	<0.001
Smoking, *n* (%) ^#^	1608 (40.52%)	685 (17.36%)	<0.001
Drinking, *n* (%) ^#^	1393 (35.11%)	651 (16.50)	<0.001
FBG, mmol/L^*^	13.34 ± 3.92	6.27 ± 2.41	<0.001
TG, mmol/L^*^	1.57 ± 0.97	1.50 ± 1.14	0.005
TC, mmol/L^*^	6.26 ± 3.63	4.47 ± 1.17	<0.001
UA, μmol/L^*^	286.95 ± 72.11	276.05 ± 100.72	0.001
HDL-C, mmol/L^*^	2.00 ± 1.36	2.08 ± 1.44	0.009
LDL-C, mmol/L^*^	3.33 ± 0.98	2.76 ± 0.97	0.001
SCr, μmol/L	83.42 ± 19.26	82.65 ± 28.73	0.165
ALT, U/L^*^	18.84 ± 8.99	18.33 ± 7.57	0.007
ADA, U/L^*^	10.10 ± 3.72	11.07 ± 2.85	<0.001
Male, U/L^*^	9.74 ± 3.65	10.85 ± 2.80	<0.001
Female, U/L^*^	10.92 ± 3.74	11.33 ± 2.89	0.001
Single-diseased vessels, *n* (%) ^#^	536 (13.51%)	−	
Double-diseased vessels, *n* (%) ^#^	313 (7.89%)	−	
Triple-diseased vessels, *n* (%) ^#^	1290 (32.51%)	−	

^#^Categorical variables are expressed as percentages. *p* values of the categorical variables were calculated by χ2 test. ^*^Continuous variables are expressed as the mean ± SD. *p* values of the continuous variables were calculated using unpaired *t* test. Abbreviations: ADA: adenosine deaminase; ALT: alanine aminotransferase; BMI: body mass index; FBG: fasting blood glucose; HDL-C: high-density lipoprotein cholesterol; LDL-C: low-density lipoprotein cholesterol; SCr: Serum creatinine; SD: standard deviation; TC: total cholesterol; TG: triglyceride; UA: uric acid.

**Figure 3:**
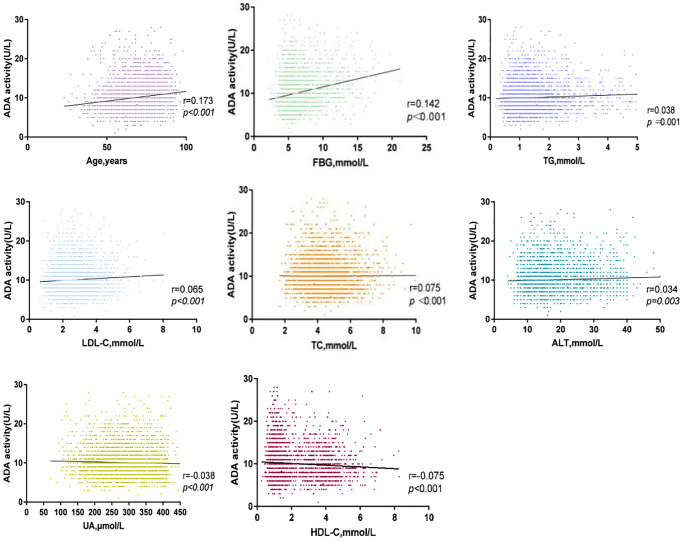
Correlation between serum ADA and TC, ALT, FBG, LDL-C, Age, UA, HDL-C, TG in ACI group.

We found that serum ADA activity was positively correlated with age (r = 0.173, *P* < 0.001), FBG (r = 0.142, *P* < 0.001), TG (r = 0.038, *P* = 0.001), LDL-C (r = 0.065, *P* < 0.001), TC (r = 0.075, *P* < 0.001) and ALT (r = 0.034, *P* = 0.003) levels in patients with ACI. Furthermore, we observed a negative relationship in UA (r = −0.038, *P* < 0.001), and HDL-C (r = −0.075, *P* < 0.001) with serum ADA. These results are shown in Figure 3.

We observed that ADA concentrations of ACI patients were affected by DM, hypertension, high alcohol consumption and smoking status. This study showed that in the experimental group, DM and hypertension markedly elevated serum ADA concentrations, whereas alcohol consumption, and smoking decreased ADA activity (Table 2).

**Table 2:** Clinical parameters and ADA activity.

	**ACI patients with**							
	**Smoking**	**Non-smoking**	**Drinking**	**Non-drinking**	**Hypertension**	**Non-hypertension**	**Diabetes**	**Non-diabetes**
Patients number, *n*	1608	2360	1393	2575	2570	1395	1087	2880
ADA activity, U/L	9.68 ± 3.57	10.39 ± 3.79	9.61 ± 3.75	10.56 ± 3.85	10.11 ± 3.62	10.08 ± 3.90	10.95 ± 3.96	9.78 ± 3.57
	0.037		0.043		0.007		>0.001	

ADA is expressed as the mean ± SD. ADA: adenosine deaminase; ACI: acute cerebral infarction; SD: standard deviation.

In our retrospective study, serum ADA concentrations of ACI patients were markedly lower compared to that in the control group (10.10 ± 3.72 vs. 11.07 ± 2.85 U/L, *P* < 0.001) (Table 1). After adjusting the multivariate logistic regression model adjusted for potential risk factors, such as BMI, FBG, TG, TC, HDL-C, LDL-C, UA, ALT, smoking, alcohol consumption, hypertension, and DM status, serum ADA concentrations were significantly associated with the presence of ACI (OR = 1.161, 95% CI: 1.140–1.183, *p* < 0.001) (Table 3).

**Table 3:** Associations between serum ADA activity and presence of stroke.

**Adjustment**	**Models**	**OR 95% CI**	***P***
**Model 1**	Crude, no adjustment	−	*P* < 0.001
**Model 2**	Adjusting for age, sex, BMI, smoking, drinking, hypertension and diabetes statues	1.144 (1.126–1.162)	*P* < 0.001
**Model 3**	Adjusting for FBG, TG, TC, HDL-C, LDL-C, UA, SCr and ALT	1.165 (1.144–1.187)	*P* < 0.001
**Model 4**	Adjusting for FBG, TG, TC, HDL-C, LDL-C, UA, ALT, age, sex, BMI, smoking, drinking, hypertension and diabetes statues	1.161 (1.140–1.183)	*P* < 0.001

Abbreviations: ADA: adenosine deaminase; ALT: alanine aminotransferase; BMI: body mass index; ACI: acute cerebral infarct; CI: confidence interval; FBG: fasting blood glucose; HDL-C: high-density lipoprotein cholesterol; LDL-C: low-density lipoprotein cholesterol; OR: odds ratio; SCr: serum creatinine; TC: total cholesterol; TG: triglyceride; UA: uric acid.

## DISCUSSION

The present study was the first to identify an independent correlation between the attenuated concentrations of serum ADA activity and the occurrence of ACI. However, ADA activity across ICAS groups defined by the number of stenotic arteries did not show significant differences.

Our results showed that serum ADA concentrations were positively correlated with TG, LDL-C, and TC levels in the ACI group. Atherosclerosis is the most common risk factor for ACI. It has been reported that purinergic signal transduction is to participate in the regulatory process of vascular inflammation and atherosclerosis and plays a key role [14]. Increasing ADA concentrations have been reported to precede macrophage accumulation of macrophages and vascular lipid deposition, and then increased with higher plaque formation in a mouse model of atherosclerosis [15]. Therefore, increased vascular ADA activity has been proposed as an early marker and trigger of atherosclerosis. Kutryb Zajac et al. [16] revealed there was a positively correlated that markers of endothelial activation, vascular lipid content, plasma triglycerides, and LDL-C.

HDL-C has long been considered good cholesterol and epidemiological studies have shown that its plasma level is negatively correlated with cardiovascular and cerebrovascular risk [17]. We also observed an inverse correlation between HDL-C levels and ADA. ADA is involved in the atherosclerosis process, and HDL-C acts as a protective factor in atherosclerosis. However, how HDL-C acts on ADA remain unclear, and further research is needed.

In this work, DM was also observed to significantly increase ADA activity in stroke patients. A study showed that ADA concentrations of subjects with DM were significantly elevated than that in healthy controls and ADA concentrations of DM subjects were positively correlated with FBG levels [18]. Previous studies have shown that diabetes is a chronic low-grade inflammatory disease in which T lymphocytes are to participate in the immune response, characterized by islet β-cell dysfunction [19, 20]. ADA not only acts as a key factor in the proliferation, differentiation, and maturation of T lymphocytes [21, 22], but it is also an important enzyme that regulates the adenosine concentrations inactivation—which also acts as an important factor in glucose and insulin homeostasis and the pathophysiology of diabetes [23–25]. Elevated ADA activity in diabetic patients accelerates adenosine decomposition, thereby influencing blood glucose homeostasis [20]. On the other hand, ADA binds to DDPIV/CD26, a transmembrane glycoprotein on the surface of T lymphocytes, via the A2b receptor, thus inhibiting glucagon-like peptide-1 (GLP-1) [26]. It acts as a key factor in promoting insulin secretion, inhibiting glucagon secretion, and promoting islet cell proliferation and differentiation of islet cells [27].

In this study, we also observed a significant effect of smoking on serum ADA activity in patients with ACI. The main components of cigarettes are nicotine, tar, carbon monoxide (CO), and other substances [28]. Nicotine in cigarettes affects the cardiovascular system through sympathetic activation [28]. Studies have shown that patients who smoke can secrete more adrenaline, which induces platelet aggregation, and CO in smoke leads to tissue ischemia and hypoxia. Adenosine inhibits platelet aggregation, dilates blood vessels, and alleviates ischemia and hypoxia. Under the condition of smoking, ADA activity through feedback adjustment, reduce the decomposition of adenosine, thus maintaining high adenosine activity to protect the cardiovascular system. Alcohol consumption exerts a negative influence on serum ADA activity.

**Figure 4:**
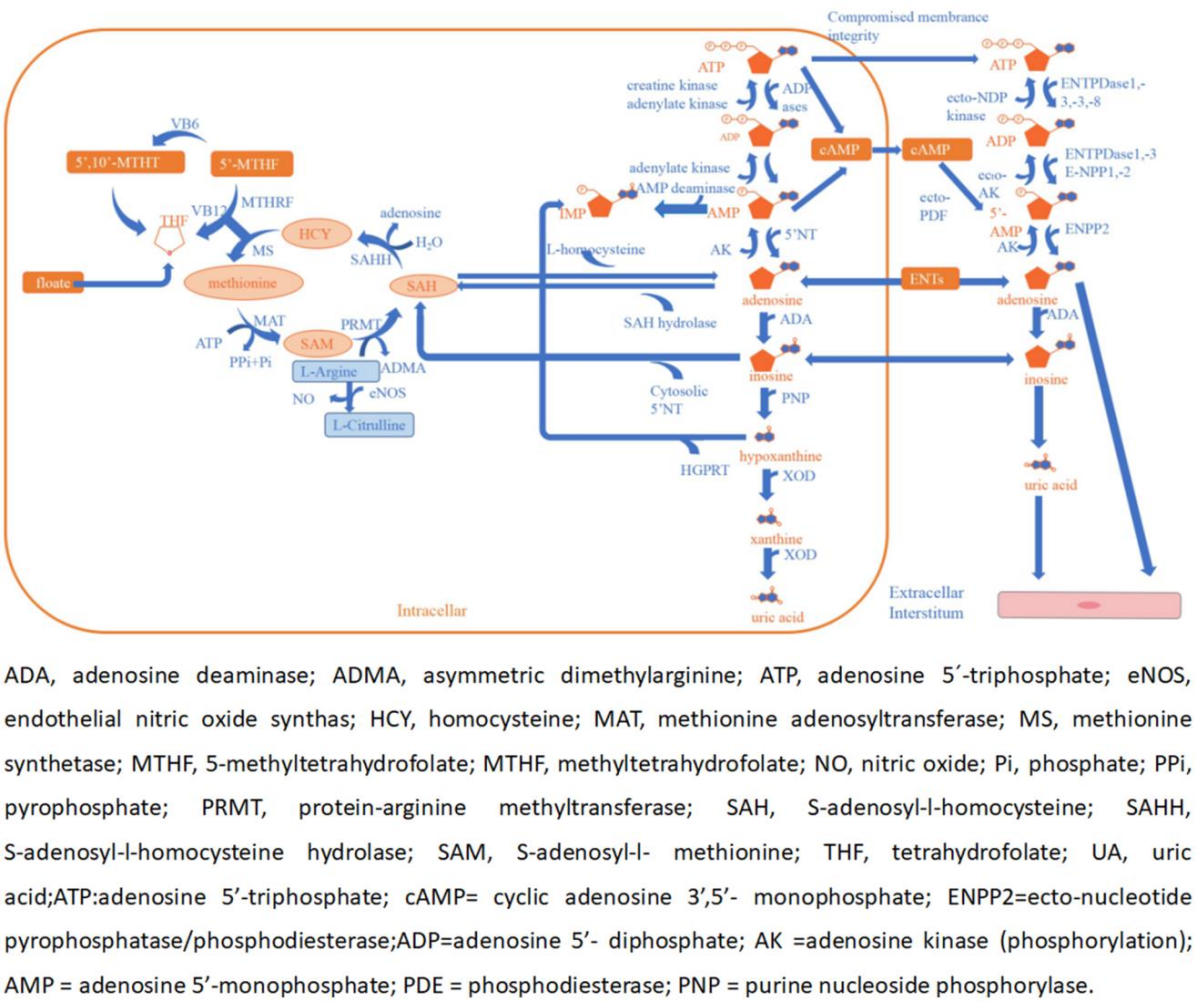
Adenosine and methionine metabolism pathways and endothelial dysfunction.

In a previous study, several metabolites were found to be risk factors for ACI such as homocysteine (Hcy), asymmetric dimethylarginine (ADMA), and UA [29–31]. However, both adenosine and NO have potential roles as an endogenous neuroprotective agent in ischemia [32–34]. Adenosine is a signaling molecule that appears in the extracellular environment and plays a key role in human physiology. It has a dual function, acting both as a homeostatic transcellular messenger and as a neuromodulator. Previous studies have indicated that extracellular adenosine concentrations can increase from approximately 1 mM at normal baseline levels to 100 mM or more during and after episodes of hypoxia or ischemia attacks [32]. It cannot pass freely through cell membranes, but it can be transported into the cell by equilibrative nucleoside transporters (ENTs) and requires the use of nucleoside transporters to facilitate this process [35]. When adenosine levels are increased rapidly, it is transported to vascular endothelial cells and red blood cells, where ADA rapidly metabolizes it to inosine [36]. ADA is a purine metabolic enzyme that irreversibly converts adenosine to inosine [37]. Adenosine can appear in the extracellular environment. Adenosine, also one of the methionine cycle products, is opposite to UA, Hcy, and ADMA in maintaining physiological homeostasis [38–40]. Furthermore, there are many enzymes, factors, and substances involved in the cycle, including L-arginine, vitamin B12, vitamin B6, and folate. Ultimately, adenosine and NO act on the cerebrovascular endothelium to induce vasodilation and also play a role in neuroprotection [33, 41, 42] (Figure 4).

The underlying mechanism of attenuated ADA activity in patients with ACI is unclear, but we believe that a complex metabolic process is involved. ADA is a key enzyme that is related to cellular metabolism and its activity, biosynthesis, and catabolism and is regulated by the neurohormone axis. When ACI occurs, adenosine activity increases rapidly, acting on endothelial cells and causing vasodilation and neuroprotection effects. Under these conditions, to maintain higher adenosine activity to protect the cerebrovascular system. ADA activity undergoes negative feedback regulation and reduces adenosine decomposition. However, this study is preliminary, and the specific mechanisms involved need to be further studied.

This study has several limitations. First, we used 3D MRA to assess intracranial arterial stenosis, but MRA is not the gold standard for assessing intracranial stenosis. Second, in this study, not all control participants underwent exhaustive MRA. Third, the ADA activity exam was a one-time exam, which may not effectively represent the fluctuation of mediator levels. Fourth, in this study, only the differences in total ADA were observed, and the differences in its isoenzymes ADA1 and ADA2 between the study group and the control group were not further evaluated. Elevated serum isoenzyme ADA2 is commonly found in viral diseases, such as immunodeficiency virus infections. Studies have detected elevated levels of tADA and its isoenzymes ADA1 and ADA2 in saliva in patients diagnosed and convalescent with the new coronavirus disease 2019 (COVID-19). Finally, our study did not evaluate interactions with oral medications that may have affected ADA levels.

## CONCLUSIONS

Our study provides proof that ADA concentrations are decreased in patients with ACI. We also show that ADA is influenced by hypertension, diabetes, and lifestyle. The results suggest that the decreased ADA activity may be involved in the pathogenesis of ACI. The exact physiopathological mechanism of ADA in ACI needs further study in the further.
